# A Copper-Rich Multinary Iodido Bismuthate with Cationic Ligands and Broad Red Emission

**DOI:** 10.1021/acs.chemmater.5c00306

**Published:** 2025-05-23

**Authors:** Jakob Möbs, Philip Klement, Lukas Gümbel, Paula Epure, Florian Weigend, Sangam Chatterjee, Johanna Heine

**Affiliations:** † Department of Chemistry and mar.quest|Marburg Center for Quantum Materials and Sustainable Technologies, 9377 Philipps-Universität Marburg, Hans-Meerwein-Straße, Marburg D-35043, Germany; ‡ Department of Physics, University of Oxford, Parks Road, OX1 3PU Oxford, U.K.; § Institute for Inorganic and Analytical Chemistry, 9175 Justus Liebig University Giessen, Heinrich-Buff-Ring 17, Giessen D-35392, Germany; ∥ Institute of Experimental Physics I and Center for Materials Research, Justus Liebig University Giessen, Heinrich-Buff-Ring 16, Giessen D-35392, Germany; ⊥ Institute of Quantum Materials and Technology, Karlsruhe Institute of Technologies, P.O. Box 3640, 76021 Karlsruhe, Germany

## Abstract

Lead halide perovskites and related hybrid metal halides exhibit exceptional semiconductor properties, enabling diverse applications in photovoltaics, solid-state lighting, and photocatalysis. Multinary halido metalates, combining multiple metals, offer unique opportunities to tune the optical and electronic properties of these materials for specific applications. Here, we present the synthesis and characterization of (Hpiz)_4_BiCu_4_I_11_·2MeCN (piz = piperazine), the most copper-rich molecular iodido bismuthate reported to date, featuring a Cu/Bi ratio of 4:1. It extends the “all-in-one” design concept of halido cuprates with cationic ligands to multinary systems and exhibits a low optical band gap of 1.82 eV (681 nm) and broad red photoluminescence centered at 1.69 eV (735 nm), making it a promising candidate for light-harvesting and near-infrared emission applications. Quantum chemical analyses attribute the reduced band gap to strong electronic interactions between Cu­(I) and Bi­(III). Additionally, the monometallic analogs (H_2_piz)­CuI_3_ and (H_2_piz)­Bi_2_I_8_ reveal the role of heterometallic interactions in modulating the optical properties. This study provides valuable insights into the design of copper–bismuth iodide systems, enriching the library of hybrid materials with customized semiconductor characteristics.

## Graphical abstract

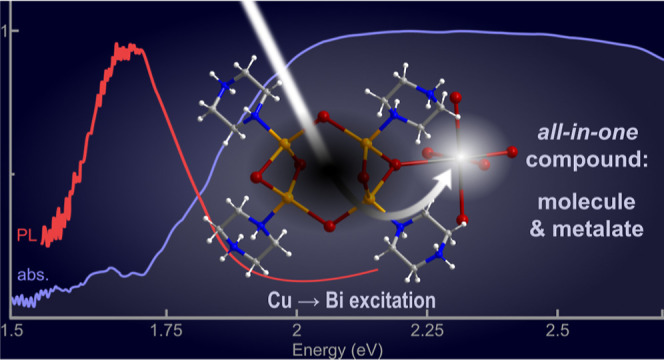

Hybrid metal halides like the prototypical perovskite (CH_3_NH_3_)­PbI_3_ have been explored as versatile semiconductor materials in the past decade.^1^ Applications include photovoltaics,^2^ solid-state lighting^3^ and photocatalysis.^4^ In an effort to broaden the scope of available materials, researchers have been investigating multinary metal halides such as the double perovskite Cs_2_AgBiBr_6_ ^5^ and layered derivatives with functional organic cations.^6^ Such multinary halido metalates featuring main group metals like Sn, Pb, Sb or Bi and transition metals like Cu or Ag combine two structurally rich halido metalate chemistries,^7–9^ leading to unprecedented anion motifs and new properties,^10–14^ although notably, rare examples featuring Pt^15^ or Hg^16^ have been prepared as well.

The community has been interested in preparing multinary iodido metalates containing Cu­(I) and Bi­(III) with particularly low optical band gaps, which makes them more suitable for light harvesting applications than the simple iodido cuprates or bismuthates which show large optical band gaps. Figure 1 summarizes anion motifs of hybrid copper iodido bismuthates with molecular anions.^17–23^ A number of copper iodido bismuthates with polymeric anions have also been reported.^24–30^

**Figure 1:**
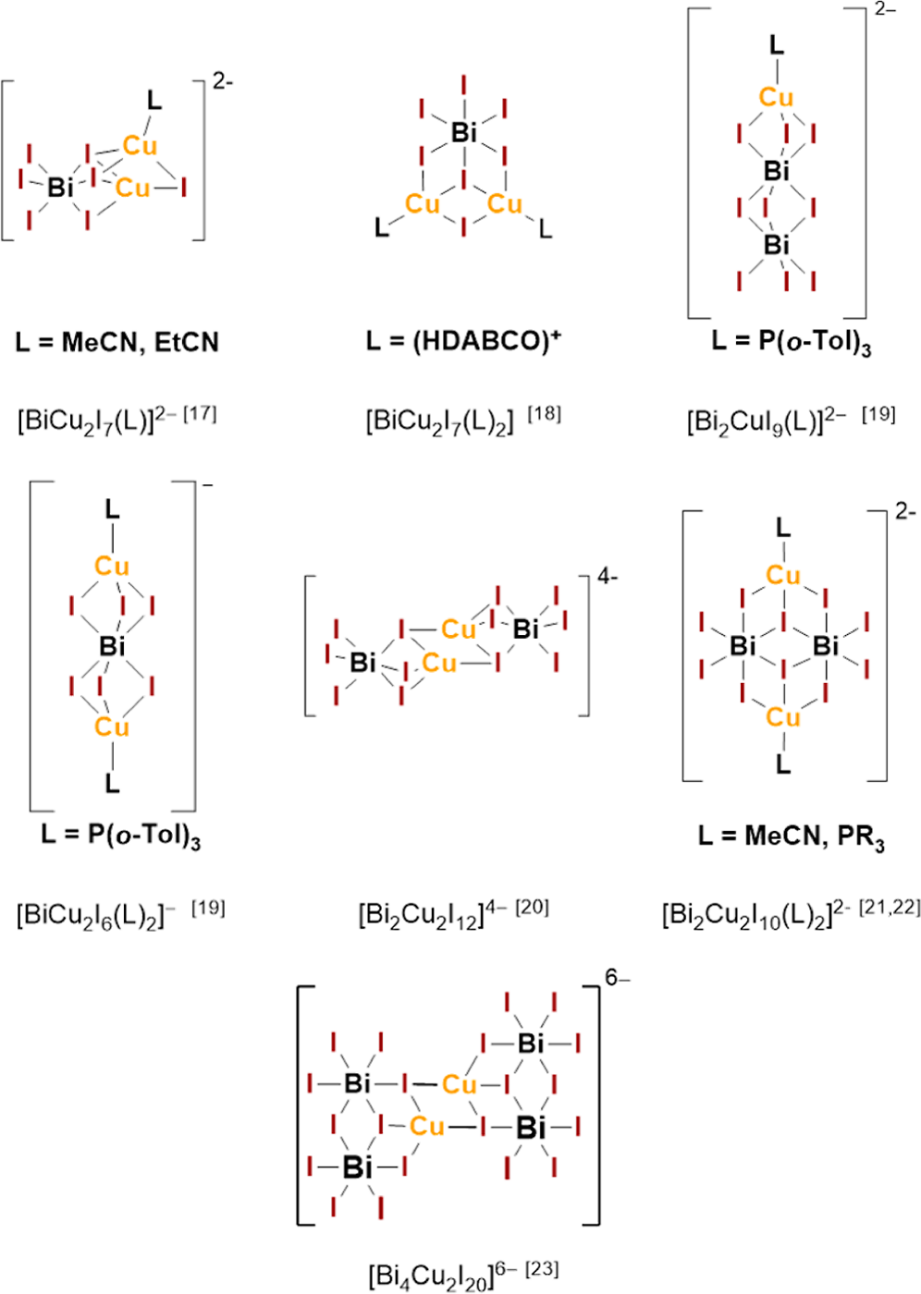
Examples of copper iodido bismuthates with molecular anions.^17–23^

Halido cuprates­(I) and copper­(I) halide compounds have been studied as efficient luminescent materials.^31^ The luminescence mechanisms in these compounds are often complex, with emissions assigned to metal- or cluster-centered transitions as well as ligand to metal charge transfer (LMCT).^31^ Additionally, self-trapped exciton emission has been observed, for example in Cs_3_Cu_2_X_5_ (X = Cl, Br, I).^32^ In contrast, investigations on the luminescence of halido bismuthates remain rare and often, only weak luminescence is observed in iodido compounds.^33,34^ In principle, metal-centered and self-trapped exciton-luminescence mechanisms are available in bismuth halide materials,^35^ but most examples feature lighter halides.^36^

Most luminescence phenomena in copper or bismuth halide materials are in the visible range of the electromagnetic spectrum. However, emission at lower energies, in the red and near-infrared region (700–1600 nm) is important for biomedical applications^37^ and telecommunication.^38^ This has been realized with a diversity of materials including rare-earth metal compounds^39^ or III–V semiconductor quantum-well heterostructures.^40^

In this work, we present the synthesis and properties of (Hpiz)_4_BiCu_4_I_11_·2MeCN (**1**, piz = piperazine), the most copper-rich copper bismuth iodide compound reported to date. **1** displays a low optical band gap of 1.82 eV (681 nm) and a broad photoluminescence band centered at 1.69 eV (735 nm). The compound can be understood as an extension of the “all-in-one” copper halide materials introduced by Li and co-workers:^41^ in this approach, the overall aggregate becomes charge-neutral by using cationic ligands coordinated to Cu^+^ ions, which avoids the typical separation into cations and anions within the compound. This can allow for unique applications such as processing into inks^42^ and use in biological systems.^43^ Additionally, we report results on the simple iodido metalates (H_2_piz)­CuI_3_ (**2**) and (H_2_piz)­Bi_2_I_8_ (**3**) to study how the materials’ optical properties change going from the homo-to the heterometallic case, while maintaining a piperazine-based organic cation.

All three compounds are prepared from stoichiometric solutions of H_2_pizI_2_ and the corresponding metal iodides in acetonitrile (cf. Synthetic Details in the Supporting Information).

**1** crystallizes in the orthorhombic space group *Cmc*2_1_ with four formula units per unit cell. The copper atoms are each tetrahedrally coordinated by three iodido- and one Hpiz^+^-ligand, forming a ring-like [(Hpiz)_4_Cu_4_I_6_]^2+^-motif. A square-pyramidal [BiI_5_]^2–^-unit is coordinated by one of the iodido ligands, making the overall coordination sphere of the bismuth atom approximately octahedral. The motif is shown in Figure 2. The Cu–N and Cu–I distances range from 2.10 Å to 2.12 Å and 2.59 Å to 2.72 Å, respectively, well in line with what is commonly observed in homo- or heterometallic iodido cuprates. With 2.93 Å to 3.15 Å the terminal Bi–I distances are in the commonly observed range as well, while with 3.38 Å, the bridging Bi–I distance is significantly elongated, but still below the sum of van der Waals radii.^19,44,45^ This is in contrast to the related neutral compound BiCuI_4_(pyridine)_5_ that features a similar Bi–I–Cu-bridge, where no Bi–I bond length elongation is observed.^46^ In the crystal, the (Hpiz)_4_BiCu_4_I_11_ molecules are ordered in a fishbone-like manner with the acetonitrile solvate molecules in small cavities in-between (see Figure 2). Surprisingly, there are no I···I or I···H contacts below the sum of the van der Waals radii connecting the molecules, which is a common feature observed in iodido metalates.^47^

**Figure 2:**
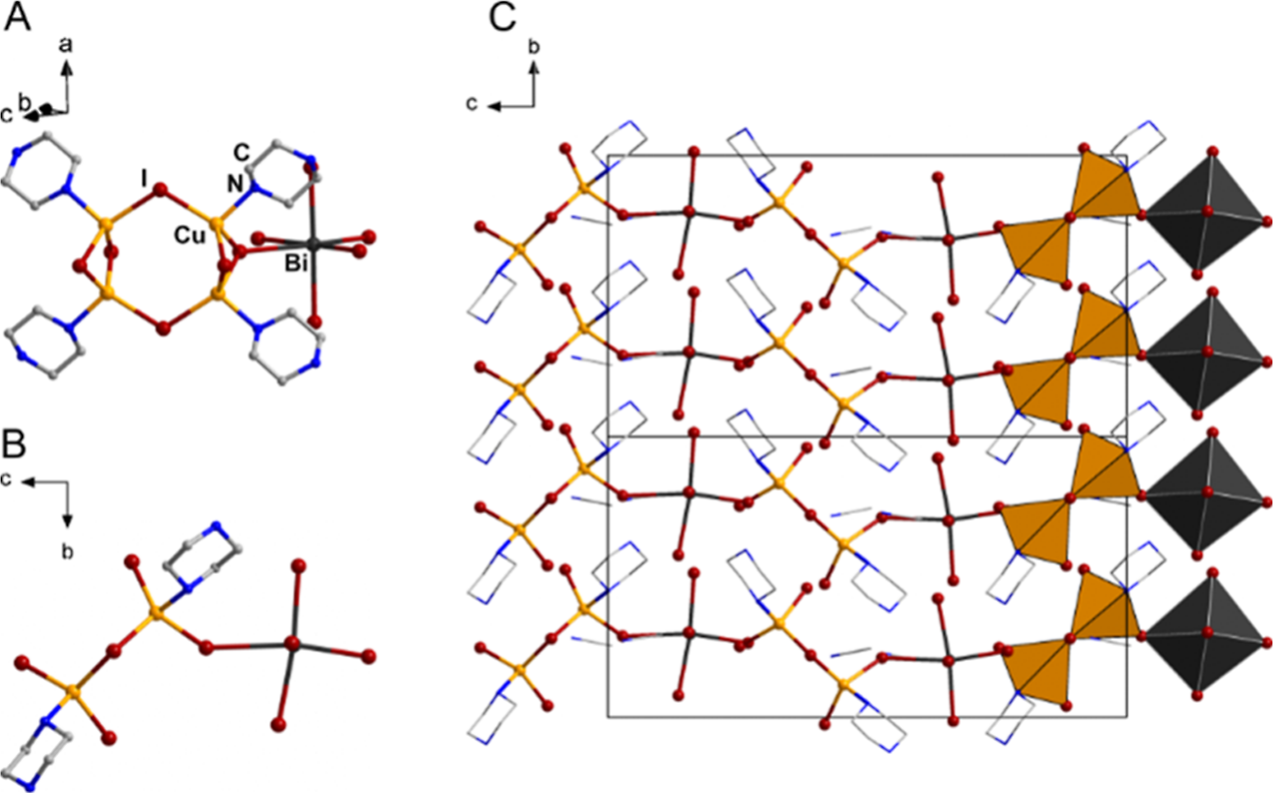
Molecular motif (A/B) and packing diagram (C) of **1**. Hydrogen atoms are omitted for clarity.

**1** features several unique aspects: The Cu to Bi ratio found here is 4:1, higher than the typical ratio found in other examples of compounds featuring Cu–I–Bi building units (see also Figure 1). A ratio of 1:5 was reported in the bismuth–rich [Bi_2_(C_4_H_8_O_3_H)_3_(C_4_H_8_O_3_H_2_)]­[CuBi_5_I_19_],^24^ and a ratio of 4:1, as in compound **1**, so far only in the complex coordination polymer HBi_3_Cu_12_I_22_(tetrahydrothiophene)_8_.^48^ The exact molecular structure found in **1** has not been observed before. However, its individual building blocks show similarities with previously reported motifs. A {(L)_4_Cu_4_I_6_}-motif has been observed before in the 2d-coordination polymer [(Et-HMTA)­Cu_4_I_6_]_*n*_, where it is interconnected into layers by an ethyl-hexaminium ligand.^49^ It can also be understood as the “double-decker”-type motif, an isomer of adamantane, previously found in organo-functionalized tetrel chalcogenides,^50^ mixed hydrido metalates^51^ and a mixed-valence bromido cuprate.^52^ Interestingly, in (Hpiz)_2_Cu_4_Br_6_ a similar motif as in **1** was reported, where two Hpiz^+^ cations on opposite sites are missing and the overall structure is distorted to allow for a trigonal-planar coordination environment for the now three-coordinated copper atoms.^53^ Similar motifs have also been observed for copper iodide compounds featuring other cationic ligands at the copper atoms.^54^ The square-pyramidal anionic [BiI_5_]^2–^ fragment found in **1** has not been reported before as an isolated molecular anion. Instead, either a trigonal-bipyramidal coordination or further aggregation toward the dinuclear [Bi_2_I_8_]^2–^ is found.^55,56^ However, similar to **1**, where a long Bi–I interaction completes the octahedral coordination sphere of the bismuth atom, compounds with chains of trans-corner-sharing {BiI_6_} octahedra have been observed.^57^

Another notable aspect is the large Bi–Cu distance found in **1**. Due to the corner-sharing connectivity between the {BiI_6_} and {CuI_3_(Hpiz)} units, it is much longer than typically observed in molecular copper iodido bismuthates (shortest Bi···Cu distance in **1**: 5.61 Å; (PPh_4_)_4_Bi_2_Cu_2_I_12_: 3.18 Å;^20^ (C_6_H_13_N_2_)_2_BiCu_2_I_7_: 3.82 Å^18^). Instead, it is closer to values found in layered anions ((C_7_H_16_N)_4_CuBiI_8_: 5.89 Å).^26^ This is remarkable with regard to the low band gap of **1** and highlights that the electronic interactions in molecular copper iodido bismuthates do not correlate with the degree of building unit condensation in a simple manner.

(H_2_piz)­CuI_3_ (**2**) crystallizes in the tetragonal space group *I*4_1_/*acd* with 16 formula units per cell. It features a chain-like anion composed of corner-sharing {CuI_4_} tetrahedra. This motif has been reported before for chlorido and bromido cuprates, as well as halogenido argentates,^58^ but not for the combination Cu/I. In **2** the anionic chains adopt a helix-like shape along the crystallographic *c*-axis with the {CuI_4_} tetrahedra following the 4_1_-screw symmetry. As with **1** the inorganic chains are quite far apart from one another with no significant I···I contacts. The chains are surrounded by [H_2_piz]^+^-ions with the NH_2_-groups pointing toward the chains. I···H_N_ distances of 2.70 Å to 2.88 Å, well below the sum of the van der Waals radii, indicate interactions between cations and anion via hydrogen bonding. Two related compounds, (H_2_piz)_2_Cu_2_I_6_·H_2_O, featuring a molecular [Cu_2_I_6_]^4–^ ion, and (H_2_piz)­Cu_2_l_4_, featuring a chain of edge-sharing {CuI_4_} tetrahedra, have been reported by Cramarossa and co-workers and main structural features are similar across all three piperazinium iodido cuprates.^59^

(H_2_piz)­Bi_2_I_8_ (**3**) crystallizes in the triclinic space group *P*1̅ with one formula unit per unit cell. The anion adopts a chain-like motif as well. It is composed of edge-sharing {BiI_6_} octahedra, which is common in polymeric iodido bismuthates.^60^ The closely related (H_2_piz)­Bi_2_I_8_·4H_2_O has been obtained by Weller and co-workers from a solvothermal reaction.^47^ In contrast to **1** and **2**, there are short I···I contacts between the individual chains of 3.92 Å to 4.07 Å, just below the double van der Waals radius of I of 4.08 Å.^44^ These connect the chains into a 3d-network. Like in **2**, the cations’ NH_2_-groups point toward the anions and connected to them by I···H_N_ contacts of 2.67 Å to 2.76 Å. The structural motifs of **2** and **3** are shown in Figure 3.

**Figure 3:**
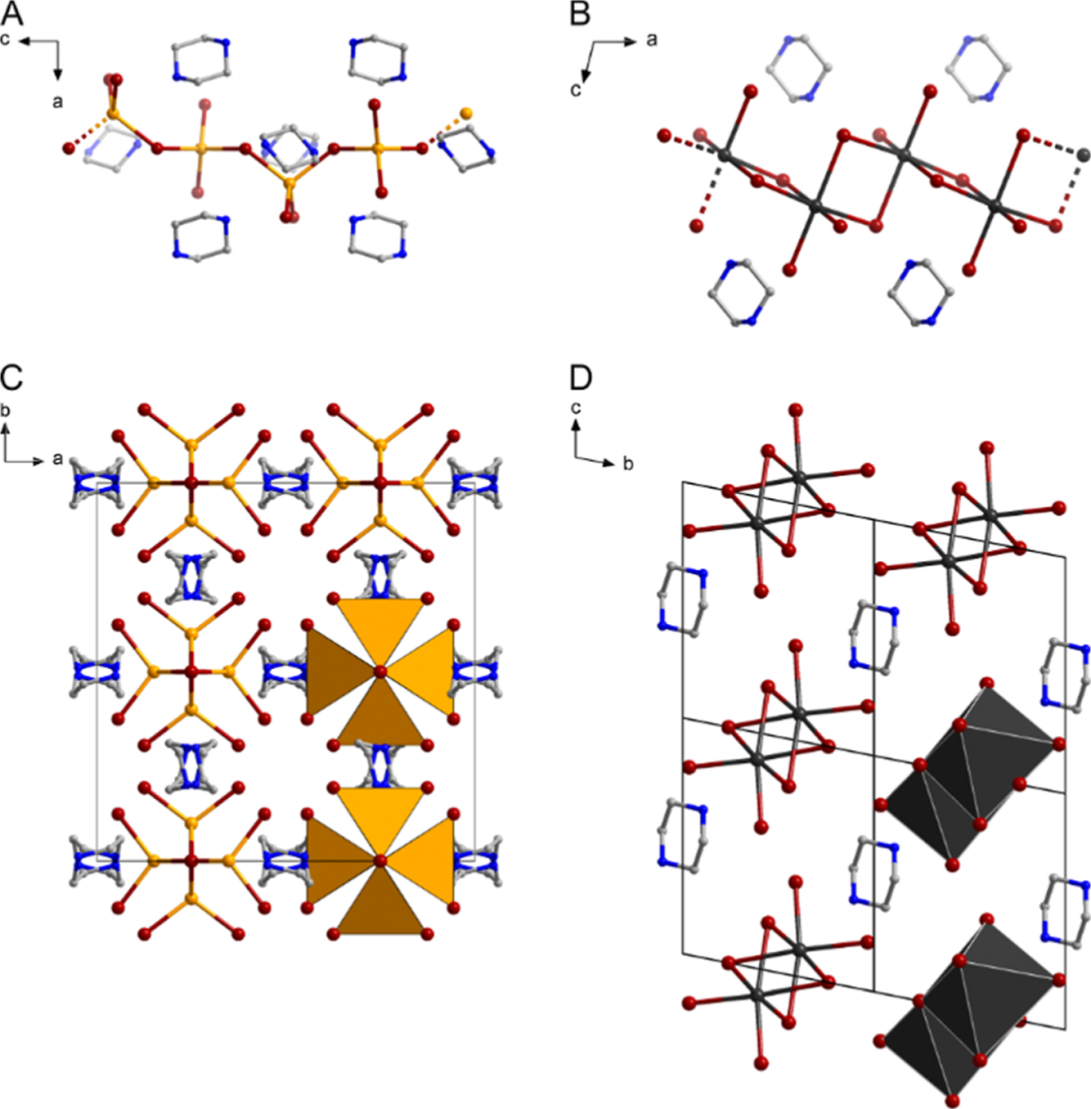
Anionic motifs and packing diagrams of **2** (A/C) and **3** (B/D). Hydrogen atoms are omitted for clarity.

All compounds have also been studied for their thermal behavior up to 1000 °C. Compound **1** is stable up to 145 °C where a first loss of mass of 3.6% occurs. This corresponds well to the amount of acetonitrile solvate molecules (3.58%). Between 185 and 255 °C the sample mass remains stable before a large and convoluted decomposition is observed between 255 and 600 °C. The remaining 40% of the sample slowly decompose further until the end of the measurement, where an amorphous residue remains. Broad endothermic peaks in the DSC data accompany all decomposition steps, but no sharp peaks indicating distinct phase transitions are observed.

Compound **2** remains stable up to 304 °C where, accompanied by a sharp endothermic peak in the DSC-data, a steep loss of mass of around 60% starts. This likely corresponds to the loss of most of the formal H_2_pizI_2_ which makes up 64% of the overall sample mass. The DSC-peak indicates a melting of the substance at the beginning of the decomposition. A second and long stretched mass loss is observed from around 600 °C until the end of the measurement. In the residue elemental copper is found next to amorphous remains.

Compound **3** is stable up to 333 °C. At this temperature a steep mass loss begins in which the sample completely decomposes. The beginning of this step is accompanied by a sharp endothermic peak in the DSC-data (onset at 333 °C, peak at 343 °C) indicating that the decomposition is initiated by the melting of the compound. We note that compared to other organic–inorganic iodido bismuthates, for example (CH_3_NH_3_)_3_Bi_2_I_9_, which decomposes at 250 °C,^61^ **3** is exceptionally stable, making it a good candidate for further investigations and potential applications. Details of the thermal measurements can be found in the Supporting Information.

Low-temperature absorption and photoluminescence measurements at 4 K yield information on the electronic processes in these compounds and the basic structure–property relationships (Figure 4). Compound **1** exhibits absorption up to the red part of the visible spectrum consistent with its red translucent appearance (Figure 4a). A Tauc analysis yields a direct band gap of 1.82 eV at 4 K, which decreases to 1.80 eV at 300 K (Figure S8). This is comparable to other copper iodido bismuthates that display band gap energies in the range of 1.6–2.1 eV.^21,27^ Furthermore, compound **1** exhibits a spectrally broad photoluminescence centered at 1.69 eV (735 nm) with a full width at half-maximum (fwhm) of 0.2 eV (90 nm). The photoluminescence is Stokes-shifted by 0.11 eV from the band gap energy. In contrast to the intensely studied double perovskite Cs_2_AgBiBr_6_ and related derivatives,^62^ reports on the luminescence properties of copper iodido bismuthates remain scarce and no clear picture has emerged with regard to expected ranges or possible luminescence mechanisms,^18,25,26^ highlighting the need for further detailed investigations to unlock potential NIR emitters.

**Figure 4:**
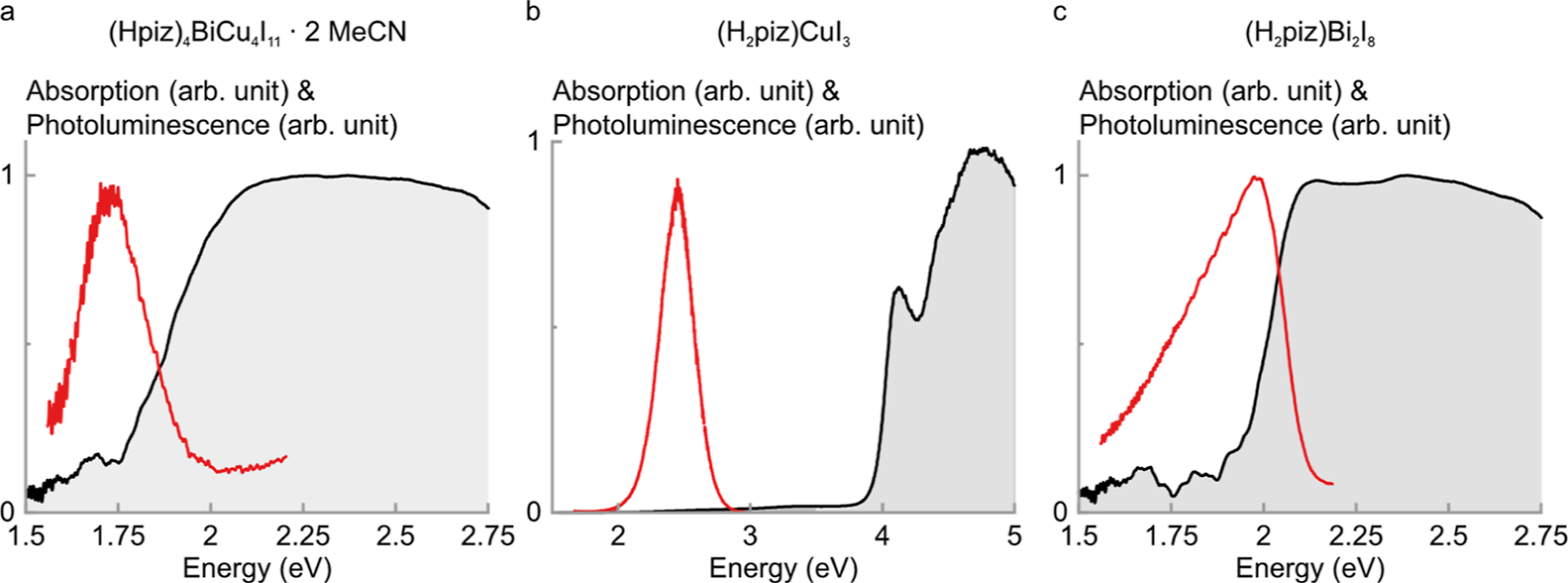
Optical properties of **1–3**. (a) Absorption and photoluminescence (PL) spectra of (Hpiz)­4BiCu4I11·2 MeCN showing a band gap energy of 1.82 eV and an emission at 1.72 eV. (b) Absorption and PL spectra of (H_2_piz)­CuI_3_ showing a band gap energy of 3.98 eV and an emission at 2.33 eV. (c) Absorption and PL spectra of (H_2_piz)­Bi_2_I_8_ showing a band gap energy of 1.98 eV and an emission at 1.97 eV.

Compound **2** exhibits absorption in the ultraviolet part of the visible spectrum consistent with its colorless opaque appearance (Figure 4b). A Tauc analysis yields a direct band gap of 3.98 eV at 300 K sample temperature. It can be concluded that the incorporation of Bi in compound **1** shifts the band gap energy lower on the order of 2 eV compared to the copper iodide compound **2**. Generally, broad ranges have been observed for the onset of absorption and for the photoluminescence peak in iodido cuprates and copper iodide-based materials.^41,63^ The values observed for **2** are, for example, comparable to those reported for (C_8_H_14_N_2_)­CuI_3_ and Cs_3_Cu_2_I_5_.^64,65^ Furthermore, **2** exhibits a bright and broad photoluminescence centered at 2.33 eV (532 nm) with a fwhm of 0.3 eV (90 nm). The photoluminescence is Stokes-shifted by as much as 1.57 eV from the band gap energy. The photoluminescence intensity is approximately 4 orders of magnitude larger compared to compound **1** when normalized for excitation photon densities and integration times indicating efficient light emission in this material, similar to other copper-based halides such as the already mentioned (C_8_H_14_N_2_)­CuI_3_.^65^ Such broad photoluminescence with large Stokes shifts occur in low-dimensional hybrid metalates and indicate strong electron–phonon coupling with consequent exciton self-trapping.^66^ A photogenerated exciton causes a local transient deformation of the crystal lattice with the ensuing deformation potential trapping the exciton.

Compound **3** exhibits absorption up to the red part of the visible spectrum and a red translucent appearance (Figure 4c). A Tauc analysis yields a direct band gap of 1.98 eV at 4 K sample temperature, which decreases to 1.96 eV at 300 K (Figure S8). This band gap energy is higher than that of the mixed copper iodido bismuthate in compound **1** indicating a good match between Bi and Cu leading to a lower band gap energy similar to the case of mixed group 14–15 metalates.^67^ The band gap energy in **1** appears typical for chain-like anions as it is similar to other iodido bismuthates.^47^ Furthermore, compound **3** exhibits a spectrally broad photoluminescence centered at 1.95 eV (636 nm) with a full width at half-maximum (fwhm) of 0.3 eV (100 nm). The photoluminescence is Stokes-shifted by 0.03 eV from the band gap energy. The peak is asymmetric with a distinct low-energy tail that could indicate the presence of defect states in the material with a broad distribution of states involved in the emission process. We exclude exciton self-trapping as the origin as the Stokes shift is small. While optical band gaps of many iodido bismuthates have been documented, leading to the emergence of a coherent picture of how the anion motif can influence the onset of absorption, only a few reports on photoluminescence in these materials are available. These are mostly focused on the composition A_3_Bi_2_I_9_ (with A = Cs, CH_3_NH_3_).^34,61^

The absorption in (Hpiz)_4_BiCu_4_I_11_ was investigated for the X-ray structure with time-dependent density functional theory^68^ employing the hybrid functional PBE0 ^69^ and polarized triple-ζ bases x2c-TZVPall-2c,^70^ using the conductor-like screening model^71^ within the all-electron relativistic exact two-component decoupling method^72^ (X2C), including as well as excluding spin–orbit coupling (SOC). For both variants, the lowest excitations and the resulting simulated spectrum as well as the difference of the (nonrelaxed) density of the first band of excited states and the ground state^73^ are shown in Figure 5. With SOC, the experimental value for the onset of excitations (∼1.8 eV) is reasonably well reproduced (2.1 eV), without, it is significantly too high (2.9 eV). Nevertheless, both variants clearly indicate that the lowest excitations involve a charge transfer from the Cu­(d)-orbitals to an (antibonding) Bi­(p)–I­(p) orbital/spinor at the BiI_6_ unit, which is the lowest unoccupied molecular orbital (LUMO) of the entire system. This excitation pathway is essentially the same as observed for other copper iodido bismuthates with much lower Cu/Bi ratios and significantly shorter Cu–Bi distances.^19^ It can be understood as a combination of the pathways found for monometallic iodido cuprates and bismuthates: The nature of the HOMO/VBM of the multinary complexes is similar to that of monometallic cuprates, while the LUMO/CBM resembles that of monometallic bismuthates. For the cuprates, generally, a metal-halide-to-ligand (MXLCT) or cluster-centered charge transfer (CCCT) is observed, with the VBM mainly being made up of Cu­(d)- and I­(p)-orbitals and the VBM consisting of combinations of Cu­(d)/I­(p)/ligand-orbitals (MXLCT) or Cu(s)/I­(p)-orbitals (CCCT).^74–76^ For the bismuthates on the other hand nonbonding I­(p) orbitals are the main contributors to the VBM, while antibonding combinations of Bi­(p)- and I­(p)-orbitals form the CBM.^77,78^

**Figure 5:**
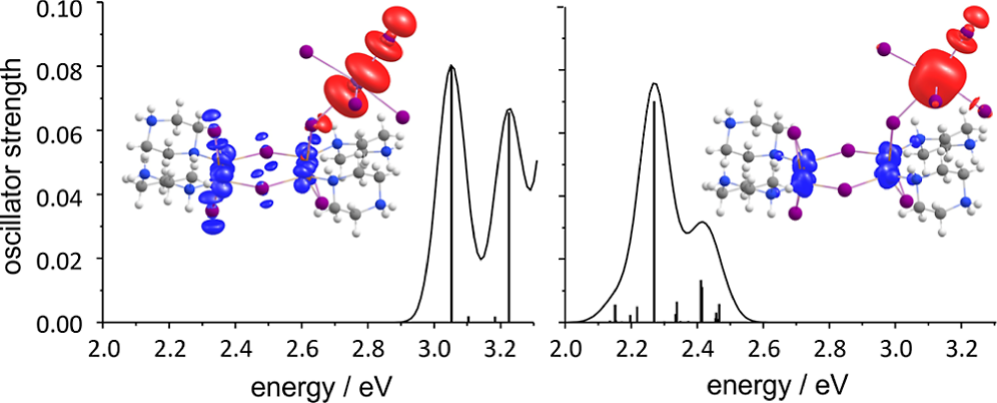
Calculated lowest excitations and simulated spectra of (Hpiz)_4_BiCu_4_I_11_ as well as differences of electron densities of the first band of excited states and the ground state without spin–orbit coupling (left) and with spin–orbit coupling (right). Blue color indicates a surplus of density for the ground state, red color for the excited state. Contours are drawn at ±0.001 au.

Taking a more detailed look at the electronic structure of **1** it is evident that the lower excitation energy with SOC included is a consequence of the lower energy of the LUMO due to spin–orbit coupling (−2.4 eV with SOC, −1.7 without; the energy of the highest occupied orbital is −5.2 eV in both cases). This, in turn, is a consequence of the splitting of the six (spin)­orbitals of the atomic 6p shell into two subshells by SOC, one of which contains two 6p_1/2_ spinors that are energetically lower than the 6p orbitals (without SOC), and the other contains four 6p_3/2_ spinors that are energetically higher than the 6p orbitals. The 6p orbital is involved in the LUMO when SOC is neglected, but the two energetically lower 6p_1/2_ spinors are involved when it is included, resulting in a lower LUMO energy and, ultimately, a lower excitation energy. Note that the shape of atomic 6p_1/2_ spinors (i.e., with SOC) is quite different from that of 6p orbitals (i.e., without SOC),^79^ which is also evident from the differences for neglecting/regarding SOC in the difference density plots in Figure 5.

## Conclusions

We have presented the synthesis and properties of a multinary all-in-one metal halide compound, (Hpiz)_4_BiCu_4_I_11_ (**1**, piz = piperazine) In comparison with the monometallic (H_2_piz)­CuI_3_ (**2**) and (H_2_piz)­Bi_2_I_8_ (**3**), **1** features a low optical band gap due to a strong electronic interaction between copper and bismuth, as well as a broad red emission. The properties and the electronic nature of the excitation are very similar to other copper iodido bismuthates, thus showing that the basic electronic structure of these compounds is quite robust toward even stark changes in composition and structural arrangement. Our results showcase that the all-in-one concept that has been broadly applied in the chemistry of copper halides can be extended toward multinary metal halides to prepare robust materials with absorption in the visible range and useful emissive properties reaching into the NIR.

## Experimental Section

### Synthesis

BiI_3_, CuI, piperazine and HI (57% solution in water, stabilizer 0.75% H_3_PO_2_) were used as supplied from commercial sources. Solvents were generally flash-distilled prior to use. For filtration cellulose filters with a pore size of 5–8 μm were used. Reactions and crystallizations were performed under inert conditions to avoid the formation of polyiodides. CHN analysis was carried out on an Elementar CHN-analyzer.

(Hpiz)_4_BiCu_4_I_11_·2 MeCN (**1**): a total of 29 mg (0.05 mmol) of BiI_3_, 38 mg (0.2 mmol) of CuI, 34 mg (0.1 mmol) of H_2_pizI_2_ and 9 mg (0.1 mmol) of piperazine were suspended in 10 mL of MeCN and heated to 95 °C under reflux cooling for 40 min. The resulting red solution with **1** suspended in it as a dark red powder was let cool to room temperature. The product was collected, washed twice with 2 mL of cold MeCN and dried at 10–3 mbar. Yield: 81 mg (71%). CHN (calculated): C 10.70 (10.49), H 2.37 (2.20), N 6.03 (6.12).

Details on the synthesis of H_2_pizI_2_, **2** and **3** are provided in the Supporting Information.

### Single Crystal Diffraction

Single crystal X-ray determination was performed on a STOE STADIVARI diffractometer with microfocus CuK radiation and a Pilatus 300 K (Dectris) detector at a temperature of 100 K Structure solution and refinement were carried out using the ShelXT and ShelXL programs,^80–82^ within the OLEX2 program suite.^83^ Cif-files have been deposited as CCDC 2420525 (**1**), 2420523 (**2**) and 2420524 (**3**). Additional details are provided in the Supporting Information (Tables S1–S3).

### Powder Diffraction

Powder patterns were recorded on a STADI MP (STOE Darmstadt) powder diffractometer with Cu Kα1 radiation with λ = 1.54056 Å at room temperature in transmission mode. The patterns confirm the presence of the respective phase determined by SCXRD measurements and the absence of any major crystalline byproducts. Patterns are shown in the Supporting Information (Figures S8–S10).

### Thermal Analysis

Thermal analysis was carried out by simultaneous TGA/DSC on a NETZSCH STA 409 C/CD in the temperature range of 25 to 1000 °C with a heating rate of 10 °C min^–1^ in a constant flow of 80 mL min^–1^ N2 Individual measurements are shown in the Supporting Information (Figures S4–S6).

### Optical Properties

Optical measurements were carried out at a low temperature of 4 K with the samples in vacuum For μ-reflectance measurements, we utilized light emitted from a tungsten lamp. The light was focused onto the sample using a 20× objective with a numerical aperture of 0.45, resulting in an approximately 250 μm spot size. The reflected light was collected by the same objective and directed into the spectrometer. To obtain reflectance spectra, we subtracted the background reflectance intensity (*R*_bg_) from the sample reflectance intensity (*R*_sample_) and normalized it using the reflectance intensity from a Semrock 350–1100 nm ultrabroadband mirror (*R*_ref_). The normalized reflectance was calculated as $R=\frac{R_{sample}-R_{bg}}{R_{ref}-R_{bg}}$ and the corresponding absorption as *A* = 1 – *R*. For compound **2** only, absorption spectra were recorded on a Varian Cary 5000 UV/vis/NIR spectrometer in the range of 400–800 nm in diffuse reflectance employing a Praying Mantis accessory (Harrick) with automatic baseline correction at a temperature of 300 K. The raw data was transformed from reflectance *R* to absorption *F*(*R*) according to the Kubelka–Munk function $F\left(R\right)=\frac{{\left(1-R\right)}^{2}}{2R}$ .

For μ-photoluminescence measurements, compounds 1 and 3 were excited using a 532 nm (2.33 eV) laser. The beam was focused into a 3 μm spot using a 20× objective with a numerical aperture of 0.45, and the excitation power density was 460 W cm^–2^. Compound 2 was excited using a 325 nm (3.82 eV) laser. The beam was focused into a 1.3 μm spot using a 36× objective with a numerical aperture of 0.4, and the excitation power density was 32 W cm^–2^.

Tauc plots for **1–3** are shown in the Figure S7.

### Computational Investigations

Calculations were done for the X-ray structure with TURBOMOLE^84^ with time-dependent density functional theory^68^ employing the hybrid functional PBE0 ^69^ and polarized triple-ζ bases x2c-TZVPall-2c^70^ using the conductor-like screening model^71^ within the all-electron relativistic exact two-component decoupling method^72^ (X2C), including as well as excluding spin–orbit coupling (SOC). For both variants, the lowest excitations and the resulting simulated spectrum as well as the difference of the (nonrelaxed) density of the first band of excited states and the ground state were obtained as described previously.^73^

## Supplementary Material



## Notes

Synthesis, additional crystallographic details, thermal analysis, powder patterns, optical properties, details of the computational investigations, cif-files corresponding to CCDC 2420523–2420525 (PDF)
