# Supplementary material for: A Copper-Rich Multinary Iodido Bismuthate with Cationic Ligands and Broad Red Emission
Source: Chem Mater. 2025 May 23;37(11):4038–46. doi: 10.1021/acs.chemmater.5c00306 (PMC12160584; doi:10.1021/acs.chemmater.5c00306)
Supplement: Supplementary file 1 [file cm5c00306_si_001.pdf]

Supporting Information on

A Copper-Rich Multinary Iodido Bismuthate with Cationic Ligands and Broad Red Emission

Jakob Möbs,[a,b,c] Philip Klement,[d] Lukas Gümbel,[d] Paula Epure,[a] Florian Weigend,[e] Sangam Chatterjee,[d] and Johanna Heine*[a].

[a] Department of Chemistry and mar.quest|Marburg Center for Quantum Materials and Sustainable Technologies, Hans-Meerwein-Straße, Marburg D-35043, Germany, E-mail: johanna.heine@chemie.uni-marburg.de

[b] Department of Physics, University of Oxford, Parks Road, OX1 3PU Oxford, United Kingdom

[c] Institute for Inorganic and Analytical Chemistry, Justus Liebig University Giessen, Heinrich-Buff-Ring 17, Giessen D-35392, Germany

[d] Institute of Experimental Physics I and Center for Materials Research, Justus Liebig University Giessen, Heinrich-Buff-Ring 16, Giessen D-35392, Germany

[e] Institute of Quantum Materials and Technology (IQMT), Karlsruhe Institute of Technologies (KIT), P.O. Box 3640, 76021 Karlsruhe, Germany

Table of contents

Synthetic Details 2

Additional crystallographic details 4

Thermal analysis 10

Optical properties 12

Powder diffraction 13

Computational investigations 15

References 15

Synthetic Details

General

BiI_3, CuI, piperazine and HI (57% solution in water, stabilizer: 0.75% H_3PO_2) were used as supplied from commercial sources. Solvents were generally flash-distilled prior to use. For filtration cellulose filters with a pore size of 5 - 8 µm were used. Reactions and crystallizations were performed under inert conditions to avoid the formation of polyiodides. CHN analysis was carried out on an Elementar CHN-analyzer.

Synthesis of H_2pizI_2

A total of 3.45 g (40 mmol) of piperazine was dissolved in 30 mL of ethanol. While cooled to 0 °C, 10.56 mL (80 mmol) of aqueous HI-solution was added dropwise. The resulting colorless powder was filtered off, washed twice with 10 mL of cold ethanol and dried at 10^{-3} mbar. Yield: 8.03 g (58.7 %). CHN (calculated): C 14.12 (14.05), H 3.61 (3.54), N 8.07 (8.19).

Synthesis of $(Hpiz)_4BiCu_4I_{11} \cdot 2$ MeCN (**1**)

A total of 29 mg (0.05 mmol) of BiI_3, 38 mg (0.2 mmol) of CuI, 34 mg (0.1 mmol) of H_2pizI_2 and 9 mg (0.1 mmol) of piperazine were suspended in 10 mL of MeCN and heated to 95 °C under reflux cooling for 40 min. The resulting red solution with **1** suspended in it as a dark red powder was let cool to room temperature. The product was collected, washed twice with 2 mL of cold MeCN and dried at 10^{-3} mbar. Yield: 81 mg (71 %). CHN (calculated): C 10.70 (10.49), H 2.37 (2.20), N 6.03 (6.12).

Synthesis of $(H_2piz)CuI_3$ (**2**)

A total of 76 mg (0.4 mmol) of CuI and 136 mg (0.4 mmol) of H_2pizI_2 were suspended in 10 mL of MeCN and heated to 95 °C under reflux cooling for 4 h. During that time **2** formed as a colourless powder in a pale yellow solution. The product was collected, while still hot, washed four times with 3 mL of cold MeCN and dried at 10^{-3} mbar. Yield: 170 mg (80 %). CHN (calculated): C 9.15 (9.02), H 2.29 (2.27), N 5.24 (5.26).

Synthesis of $(H_2piz)Bi_2I_8$ (**3**)

A total of 118 mg (0.2 mmol) of BiI_3 and 34 mg (0.1 mmol) of H_2pizI_2 were suspended in 15 mL of MeCN and heated to 95 °C under reflux cooling for 2 h. The resulting red solution with **3** suspended in it as a dark red powder was let cool to room temperature. The product was collected, washed twice with

2 mL of cold MeCN and dried at 10^{-3} mbar. Yield: 50 mg (32 %). CHN (calculated): C 3.40 (3.16), H 0.96 (0.80), N 1.79 (1.84).

Single crystals suitable for X-ray diffraction of all three compounds were grown by filtering the reaction mixture after heating while still hot and letting it sit at room temperature for several days.

Additional crystallographic details

Single crystal X-ray determination was performed on a STOE STADIVARI diffractometer with microfocus CuK$_{\alpha}$ radiation and a Pilatus 300K (Dectris) detector at a temperature of 100 K. Structure solution and refinement were carried out using the ShelXT and ShelXL programs,[1–3] within the OLEX2 program suite.[4]

Table S1: Crystallographic data for **1**, CCDC 2420525.

	**1**
Empirical formula	$C_{20}H_{50}BiCu_4I_{11}N_{10}$
Formula weight	2289.74
Temperature/K	100
Crystal system	orthorhombic
Space group	$Cmc2_1$
a/Å	13.5687(2)
b/Å	14.0411(3)
c/Å	25.9449(4)
α/°	90
β/°	90
γ/°	90
Volume/Å^3	4943.01(15)
Z	4
ρ_{calc}g/cm^3	3.077
μ/mm^{-1}	62.912
Absorption correction (T_{min}/T_{max})	0.0023 / 0.0381
F(000)	4088.0
Crystal size/mm^3	0.055 × 0.04 × 0.031
Radiation	CuKα (λ = 1.54178)
2Θ range for data collection/°	6.814 to 143.626
Index ranges	$-16 \le h \le 16$, $-17 \le k \le 16$, $-31 \le l \le 19$
Reflections collected	34726
Independent reflections	4296 [R_{int} = 0.0750, R_{sigma} = 0.0538]
Data/restraints/parameters	4296/92/251
Goodness-of-fit on F^2	1.022
Final R indexes [I>=2σ (I)]	R_1 = 0.0468, wR_2 = 0.1054
Final R indexes [all data]	R_1 = 0.0543, wR_2 = 0.1075
Largest diff. peak/hole / e Å^{-3}	1.28/-1.99
Hooft parameter	0.021(9)

Details of crystal structure measurement and refinement: The structure was refined as an inversion twin with BASF of 0.50(2). All non-hydrogen atoms were refined with anisotropic thermal displacement

parameters. Hydrogen atoms were assigned to geometrically ideal positions and included in the structure factor calculations. There is one molecule of solvate acetonitrile in the asymmetric unit, which is disordered over two position with occupancies of 54.5 % and 45.5 %. To ensure a stable refinement DFIX, DELU, SIMU and RIGU restraints, as implemented in the FragmentDB [5] needed to be applied to the disordered moiety. The checkCIF level B alert `PLAT342` on low C-C bond precision can be traced back to this moiety. Therefore, it does not indicate severe problems with the structural model.

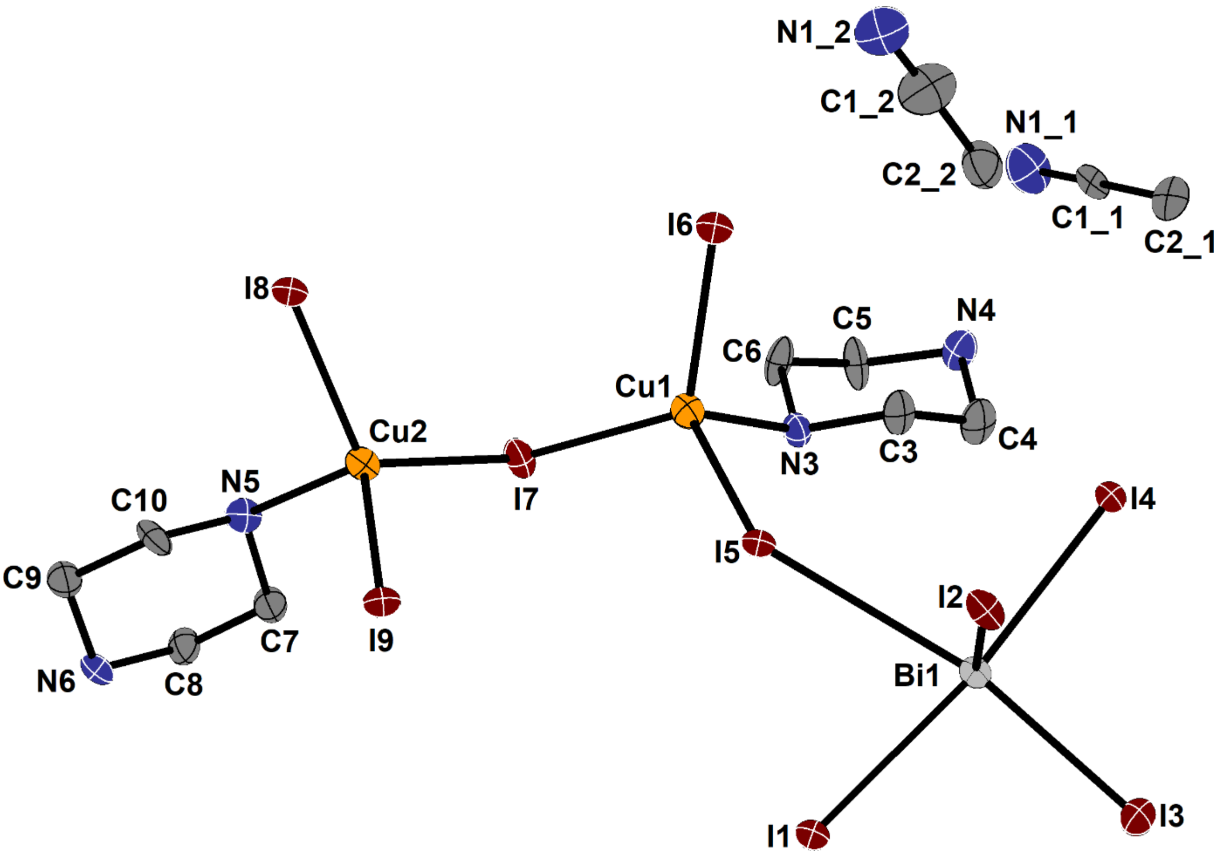

Figure S1: Asymmetric unit of **1**, ellipsoids at 50 % probability. Hydrogen atoms are omitted for clarity.

Table S2: Crystallographic data for **2**, CCDC 2420523.

	**2**
Empirical formula	$C_4H_{12}CuI_3N_2$
Formula weight	532.40
Temperature/K	100.00
Crystal system	tetragonal
Space group	$I4_1/acd$
a/Å	16.8068(11)
b/Å	16.8068(11)
c/Å	16.7844(11)
α/°	90
β/°	90
γ/°	90
Volume/Å^3	4741.1(7)
Z	16
ρ_{calc}g/cm^3	2.984
μ/mm^{-1}	63.541
Absorption correction (T_{min}/T_{max})	0.0001 / 0.0014
F(000)	3808.0
Crystal size/mm^3	0.07 × 0.05 × 0.04
Radiation	Cu Kα (λ = 1.54186)
2Θ range for data collection/°	10.528 to 144.294
Index ranges	-14 ≤ h ≤ 17, -20 ≤ k ≤ 20, -17 ≤ l ≤ 20
Reflections collected	1169
Independent reflections	1169 [R_{int}(all) = 4.12, R_{merge} = 0.226 R_{sigma} = 0.0103]
Data/restraints/parameters	1169/0/49
Goodness-of-fit on F^2	1.245
Final R indexes [I>=2σ (I)]	R_1 = 0.0524, wR_2 = 0.1497
Final R indexes [all data]	R_1 = 0.0530, wR_2 = 0.1500
Largest diff. peak/hole / e Å^{-3}	2.62/-1.1

Details of crystal structure measurement and refinement: The crystal was merohedrally twinned twice after (0 0 1 0 -1 0 1 0 0) and (-1 0 0 0 0 -1 0 -1 0) with the corresponding domains refined to 20.3(3) % and 18.0(3) % of the full crystal, respectively. After the initial solution on the full dataset, the data was detwinned using PLATON's HKLF5 generator. All non-hydrogen atoms were refined anisotropically and no restraints or constraints were needed, although a checkCIF level B alert `PLAT342` on low C-C bond precision remains, most likely due to the low data quality because of the twinning. Hydrogen atoms were assigned to geometrically ideal positions and included in the structure factor calculations. In addition to the twinning the refinement suffered from quite high X-ray

absorption of the compound, resulting in a maximum residue electron density of 2.6 eÅ^{-3} close to the iodine positions.

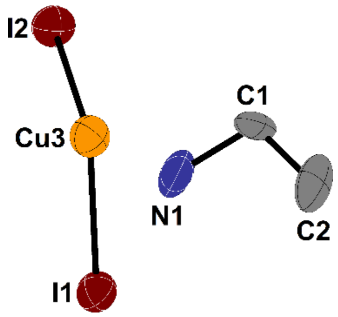

Figure S2: Asymmetric unit of **2**, ellipsoids at 50 % probability. Hydrogen atoms are omitted for clarity.

Table S3: Crystallographic data for **3**, CCDC 2420524.

	**3**
Empirical formula	$C_2H_6BiI_4N$
Formula weight	760.66
Temperature/K	100
Crystal system	triclinic
Space group	P-1
a/Å	7.5607(2)
b/Å	7.9529(2)
c/Å	9.8985(3)
α/°	100.332(2)
β/°	103.776(2)
γ/°	92.186(2)
Volume/Å^3	566.66(3)
Z	2
ρ_{calc}g/cm^3	4.458
μ/mm^{-1}	115.575
Absorption correction (T_{min}/T_{max})	0.00001 / 0.0007
F(000)	640.0
Crystal size/mm^3	0.061 × 0.025 × 0.024
Radiation	Cu Kα (λ = 1.54178)
2Θ range for data collection/°	9.378 to 134.988
Index ranges	-9 ≤ h ≤ 9, -6 ≤ k ≤ 9, -11 ≤ l ≤ 11
Reflections collected	23032
Independent reflections	2025 [R_{int} = 0.0412, R_{sigma} = 0.0143]
Data/restraints/parameters	2025/0/73
Goodness-of-fit on F^2	1.168
Final R indexes [I>=2σ (I)]	R_1 = 0.0379, wR_2 = 0.1059
Final R indexes [all data]	R_1 = 0.0421, wR_2 = 0.1079
Largest diff. peak/hole / e Å^{-3}	2.33/-2.14

Details of crystal structure measurement and refinement: All non-hydrogen atoms were refined anisotropically and no restraints or constraints were needed. Hydrogen atoms were assigned to geometrically ideal positions and included in the structure factor calculations. Due to the severe X-ray absorption of the compound (μ = 115.6 mm^{-1}) quite high minimum and maximum residue electron density peaks remain. Especially, the minimum peak of -2.05 eÅ^{-3} close to the nitrogen position (resulting in a checkCIF A level alert PLAT976) is concerning with regard to the reliability of the model. In general, these peaks are expected in the vicinity of heavy atoms, if they are artifacts of absorption. However, when looking at the structure as a whole there is little doubt about the observed motifs and the quality is more than good enough for the purpose. The only major problem is that it is not possible reliably to assign the nitrogen position to the correct atom of the piperazinium moiety based on the X-

ray data alone. As mentioned in the main manuscript, however, the occurrence of I···H contacts gives a strong hint to which position the nitrogen atom occupies.

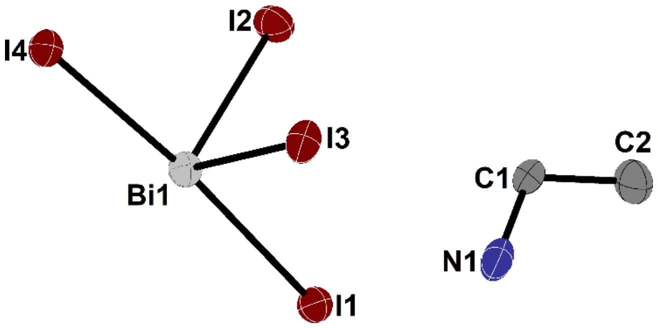

Figure S3: Asymmetric unit of **3**, ellipsoids at 50 % probability. Hydrogen atoms are omitted for clarity.

Thermal analysis

Thermal analysis was carried out by simultaneous TGA/DSC on a NETZSCH STA 409 C/CD in the temperature range of 25 °C to 1000 °C with a heating rate of 10 °C min^{-1} in a constant flow of 80 ml min^{-1} N_2.

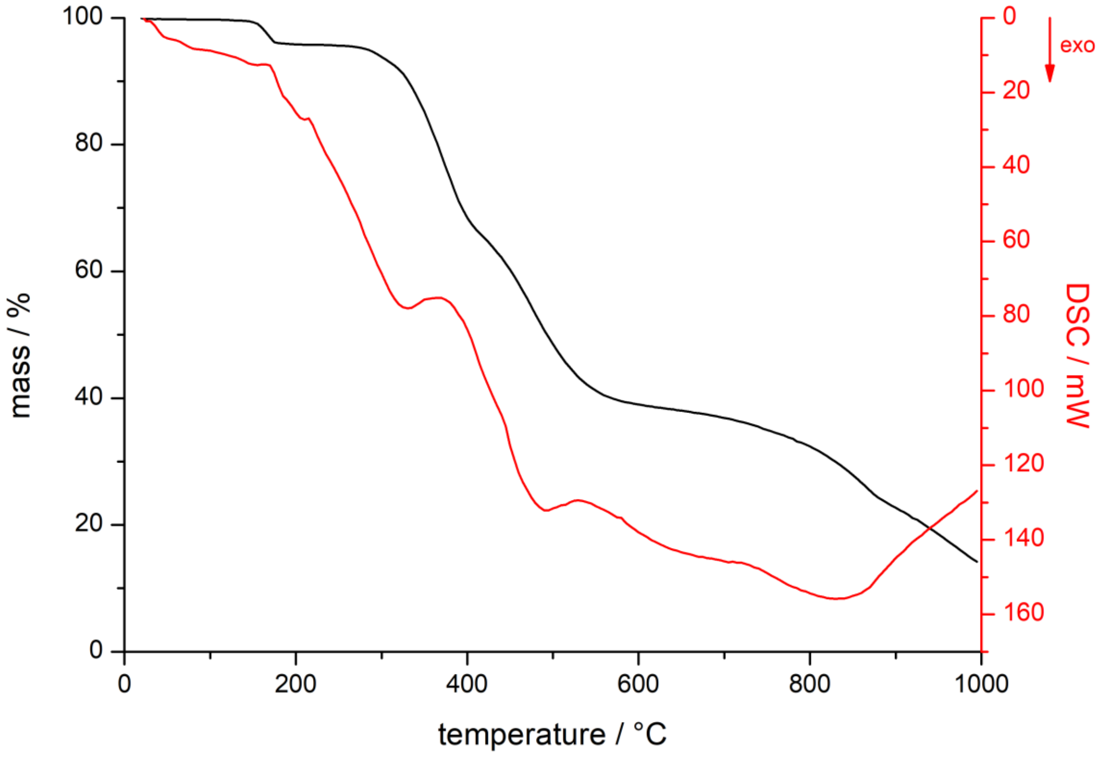

Figure S4: TGA/DSC data for **1.**

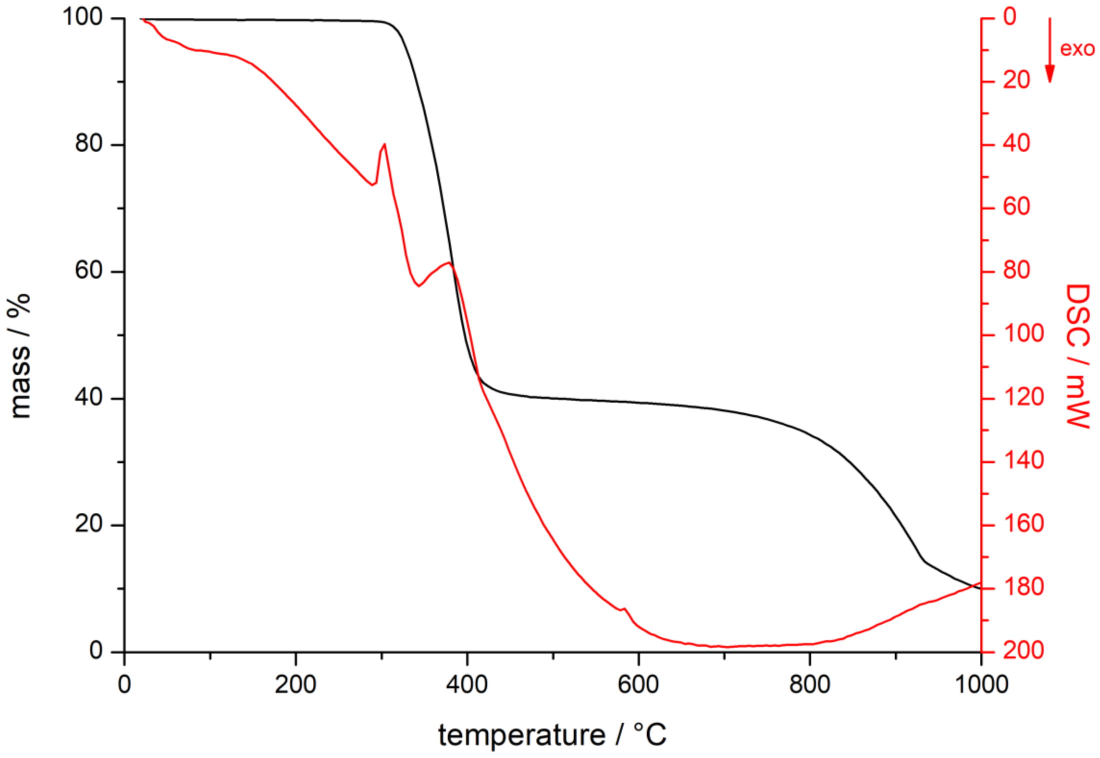

Figure S5: TGA/DSC data for **2.**

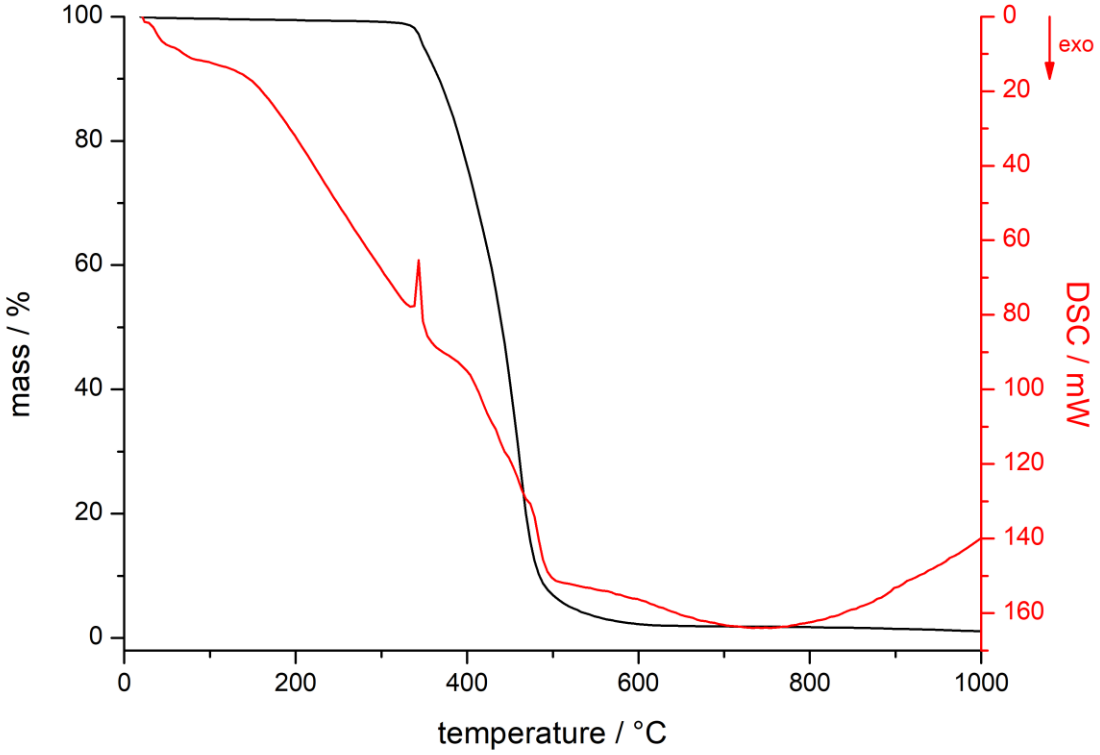

Figure S6: TGA/DSC data for **3.**

Optical properties

Optical measurements were carried out at a low temperature of 4 K with the samples in vacuum. For µ-reflectance measurements, we utilized light emitted from a tungsten lamp. The light was focused onto the sample using a 20× objective with a numerical aperture of 0.45, resulting in an approximately 250 µm spot size. The reflected light was collected by the same objective and directed into the spectrometer. To obtain reflectance spectra, we subtracted the background reflectance intensity (R_{bg}) from the sample reflectance intensity (R_{sample}) and normalized it using the reflectance intensity from a Semrock 350–1100 nm ultrabroadband mirror (R_{ref}). The normalized reflectance was calculated as $R = \frac{R_{\text{sample}} - R_{\text{bg}}}{R_{\text{ref}} - R_{\text{bg}}}$ and the corresponding absorption as $A = 1 - R$. For compound **2** only, absorption spectra were recorded on a *Varian Cary 5000* UV/Vis/NIR spectrometer in the range of 400–800 nm in diffuse reflectance employing a Praying Mantis accessory (*Harrick*) with automatic baseline correction at a temperature of 300 K. The raw data was transformed from reflectance *R* to absorption $F(R)$ according to the Kubelka-Munk function $F(R) = \frac{(1-R)^2}{2R}$.

For µ-photoluminescence measurements, compounds **1** and **3** were excited using a 532 nm (2.33 eV) laser. The beam was focused into a 3 µm spot using a 20× objective with a numerical aperture of 0.45, and the excitation power density was 460 W cm^{-2}. Compound **2** was excited using a 325 nm (3.82 eV) laser. The beam was focused into a 1.3 µm spot using a 36× objective with a numerical aperture of 0.4, and the excitation power density was 32 W cm^{-2}.

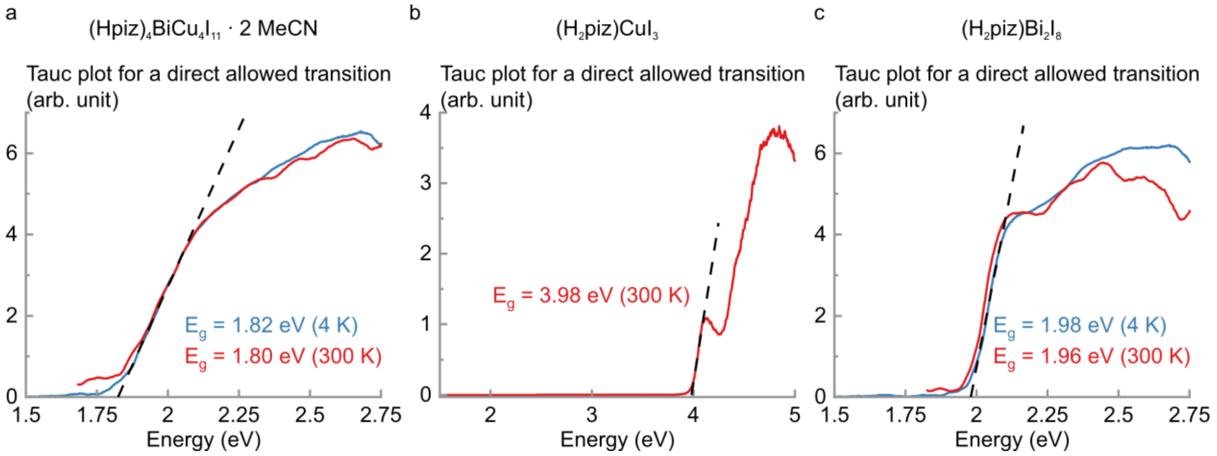

Figure S7. a) Tauc plot for a direct allowed transition for compound **1** showing a band-gap energy of 1.82 and 1.80 eV at 4 and 300 K sample temperature, respectively. b) Tauc plot for a direct allowed transition for compound **2** showing a band-gap energy of 3.98 eV at 4 K. c) Tauc plot for a direct allowed transition for compound **3** showing a band-gap energy of 1.98 and 1.96 eV at 4 and 300 K sample temperature, respectively.

Powder diffraction

Powder patterns were recorded on a *STADI MP* (*STOE* Darmstadt) powder diffractometer with $CuK_{\alpha 1}$ radiation with λ = 1.54056 Å at room temperature in transmission mode. The patterns confirm the presence of the respective phase determined by SCXRD measurements and the absence of any major crystalline by-products.

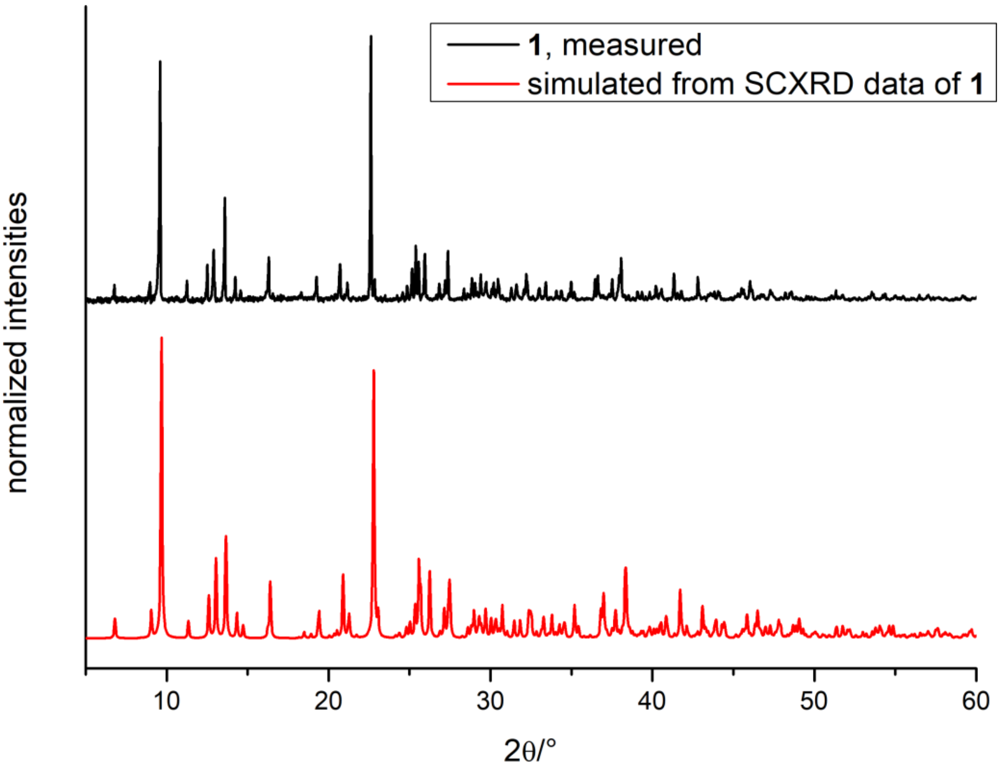

Figure S8: Measured and simulated powder diffraction patterns of **1**.

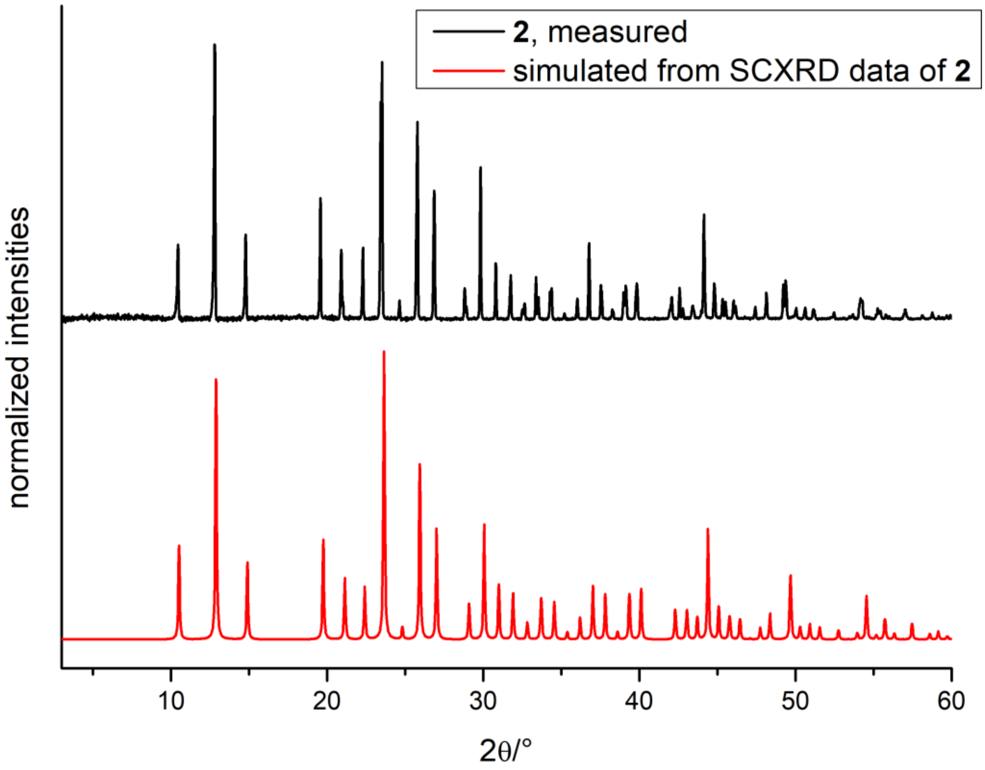

Figure S9: Measured and simulated powder diffraction patterns of **2**.

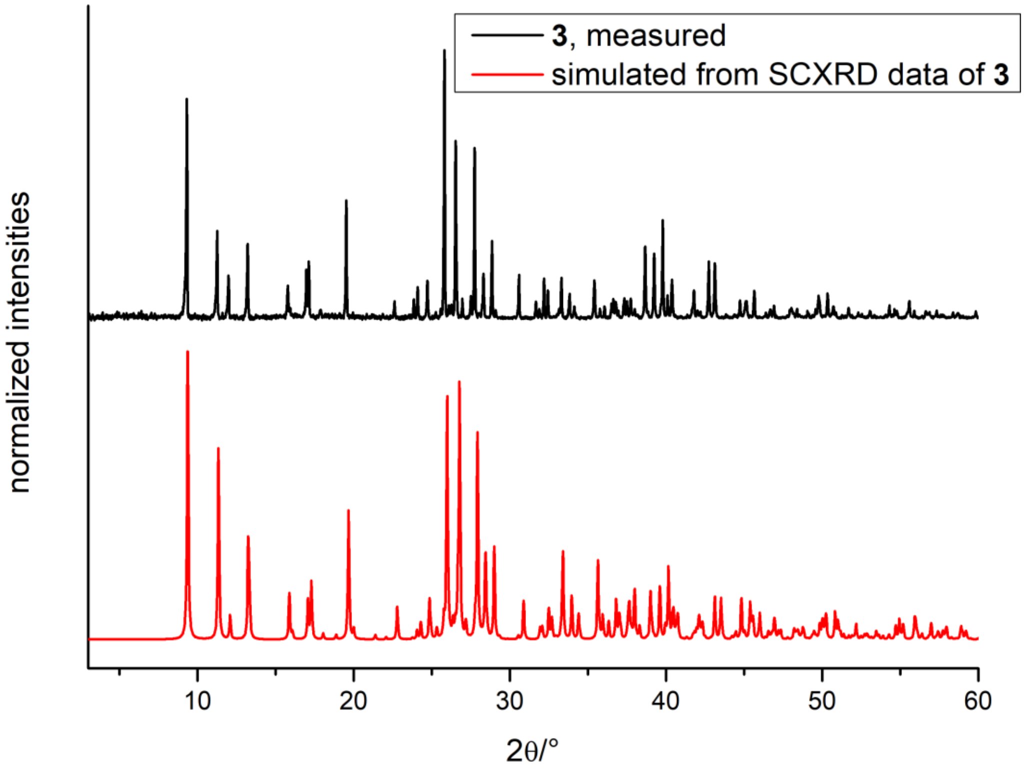

Figure S10: Measured and simulated powder diffraction patterns of **3**.

Computational investigations

Calculations were done for the X-ray structure with TURBOMOLE[6] with time-dependent density functional theory[7] employing the hybrid functional PBE0[8] and polarized triple-zeta bases x2c-TZVPall-2c[9] using the conductor-like screening model[10] within the all-electron relativistic exact two-component decoupling method[11] (X2C), including as well as excluding spin-orbit coupling (SOC). For both variants, the lowest excitations and the resulting simulated spectrum as well as the difference of the (non-relaxed) density of the first band of excited states and the ground state were obtained as described previously.[12]

References

(1) Sheldrick, G. M. A short history of SHELX. *Acta Crystallogr. A* **2008**, *64* (Pt 1), 112–122. DOI: 10.1107/S0108767307043930.

(2) Sheldrick, G. M. SHELXT - integrated space-group and crystal-structure determination. *Acta Crystallogr. A* **2015**, *71* (Pt 1), 3–8. DOI: 10.1107/S2053273314026370.

(3) Sheldrick, G. M. Crystal structure refinement with SHELXL. *Acta Crystallogr. C* **2015**, *71* (Pt 1), 3–8. DOI: 10.1107/S2053229614024218.

(4) Dolomanov, O. V.; Bourhis, L. J.; Gildea, R. J.; Howard, J. A. K.; Puschmann, H. OLEX2 : a complete structure solution, refinement and analysis program. *J. Appl. Crystallogr.* **2009**, *42* (2), 339–341. DOI: 10.1107/S0021889808042726.

(5) Kratzert, D.; Holstein, J. J.; Krossing, I. DSR: enhanced modelling and refinement of disordered structures with SHELXL. *J. Appl. Crystallogr.* **2015**, *48* (Pt 3), 933–938. DOI: 10.1107/S1600576715005580.

(6) TURBOMOLE V7.9 2024, a development of University of Karlsruhe and Forschungszentrum Karlsruhe GmbH, 1989-2007, TURBOMOLE GmbH, since 2007; available from https://www.turbomole.org.

(7) Kühn, M.; Weigend, F. Implementation of Two-Component Time-Dependent Density Functional Theory in TURBOMOLE. *J. Chem. Theory Comput.* **2013**, *9* (12), 5341–5348. DOI: 10.1021/ct400743r.

(8) Perdew, J. P.; Ernzerhof, M.; Burke, K. Rationale for mixing exact exchange with density functional approximations. *J. Chem. Phys.* **1996**, *105* (22), 9982–9985. DOI: 10.1063/1.472933.

(9) Pollak, P.; Weigend, F. Segmented Contracted Error-Consistent Basis Sets of Double- and Triple-ζ Valence Quality for One- and Two-Component Relativistic All-Electron Calculations. *J. Chem. Theory Comput.* **2017**, *13* (8), 3696–3705. DOI: 10.1021/acs.jctc.7b00593.

(10) Pausch, A. Consistent Analytical Second Derivatives of the Kohn-Sham DFT Energy in the Framework of the Conductor-Like Screening Model through Gaussian Charge Distributions. *J. Chem. Theory Comput.* **2024**, *20* (8), 3169–3183. DOI: 10.1021/acs.jctc.4c00052.

(11) Peng, D.; Middendorf, N.; Weigend, F.; Reiher, M. An efficient implementation of two-component relativistic exact-decoupling methods for large molecules. *J. Chem. Phys.* **2013**, *138* (18), 184105. DOI: 10.1063/1.4803693.

(12) Kühn, M.; Weigend, F. Phosphorescence lifetimes of organic light-emitting diodes from two-component time-dependent density functional theory. *J. Chem. Phys.* **2014**, *141* (22), 224302. DOI: 10.1063/1.4902013.
